# Supplementary material for: Engineered reproductively isolated species drive reversible population replacement
Source: Nat Commun. 2021 Jun 2;12:3281. doi: 10.1038/s41467-021-23531-z (PMC8173020; doi:10.1038/s41467-021-23531-z)
Supplement: Supplementary file 1 — Supplementary Information [file 41467_2021_23531_MOESM1_ESM.docx]

Supplementary Materials for

**Engineered Reproductively Isolated Species Drive Reversible Population Replacement**

Anna Buchman^1,*^, Isaiah Shriner^1,*^, Ting Yang^1^, Junru Liu^1^, Igor Antoshechkin^2^, John M. Marshall^3,4^, Michael W. Perry^1^ and Omar S. Akbari^1,5,†^

**Affiliations:**

^1^Section of Cell and Developmental Biology, University of California, San Diego, La Jolla, California, 92093, United States of America.

^2^Division of Biology and Biological Engineering (BBE), California Institute of Technology, Pasadena, California 91125

^3^Division of Epidemiology & Biostatistics, School of Public Health, University of California, Berkeley, CA 94720

^4^Innovative Genomics Institute, Berkeley, CA 94720

^5^Tata Institute for Genetics and Society-UCSD, La Jolla, California, USA

* equal contributions

^†^Correspondence to: Omar S. Akbari, [oakbari@ucsd.edu](mailto:oakbari@ucsd.edu)

**This PDF file includes:**

**Supplementary Figures 1-7**

**Supplementary Figure. 1.** Constructs used in this study

**Supplementary Figure. 2.** Schematic of the genetic crossing scheme used to engineer SPECIES

**Supplementary Figure. 3.** Generation of 8 synthetic SPECIES

**Supplementary Figure. 4.** Molecular characterization of protective indel mutations

**Supplementary Figure. 5.** Reproductive isolation between double-homozygous speciated lines

**Supplementary Figure. 6.** Two-Factor RNAseq Comparisons

**Supplementary Figure. 7.** Heatmap of the RNAseq Data

**Source Data File**

**Figure 1c,d.** Lethality and Rescue by targeting single and multiple genes

**Figure 2c,d.** Laying/survival experiments for SPECIES

**Figure 3d.** TPM expression analysis for genes of interest.

**Figure 4.** Gene Drive Experimental Raw Data

**Supplementary Figure 5b.** Interspecies crosses

**Supplementary Figure 6a.** WTxA vs. A deseq2 Data

**Supplementary Figure 6b.** WTxB vs. B deseq2 Data

**Supplementary Figure 6c.** WTxC vs. C deseq2 Data

**Supplementary Figure 6d.** WTxD vs. D deseq2 Data

**Supplementary Table 1.** Embryo collections for RNA Sequencing

**Supplementary Table 2.** RNAseq expression values for all genes and all 23 samples sequenced.

**Supplementary Table 3.** DEseq analysis comparing SPECIES outcrosses to SPECIES self crosses.

**Supplementary Table 4.** Gene Drive Experimental Data

**Supplementary Table 5** Primers used in this study.

**Supplementary Table 6.** Population studies with associated fitness costs of SPECIES strains relative to WT

**Supplementary Table 7.** Correlation between all RNA-seq samples.

**Supplementary Table 8.** Outcrosses to Global Diversity Lines.

**SUPPLEMENTARY FIGURES**

**
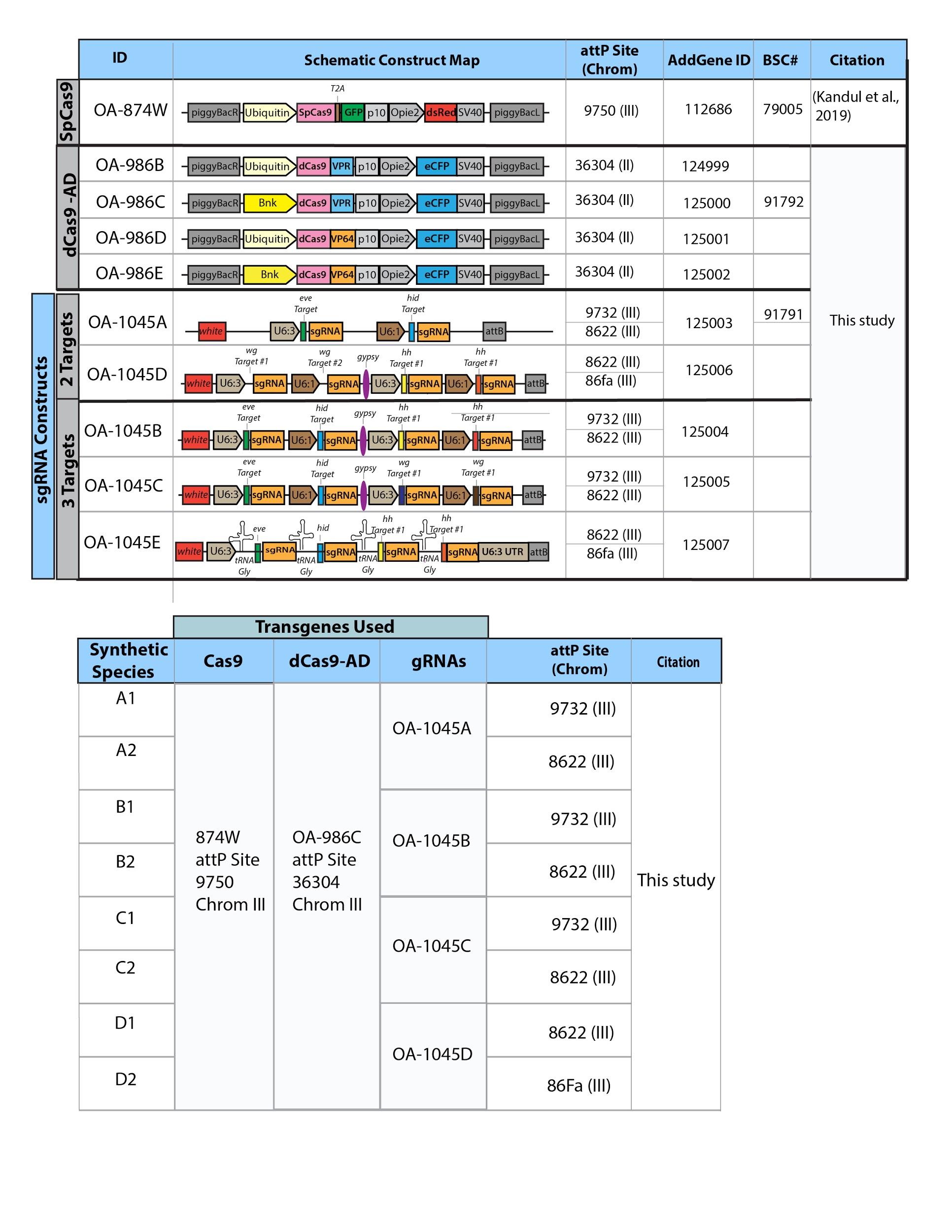
**

**Supplementary Figure 1. Constructs used in this study.** A list of constructs used in this study, providing the construct ID, construct schematics, chromosomal insertion sites, Addgene ID number, Bloomington Stock number and citation.

**
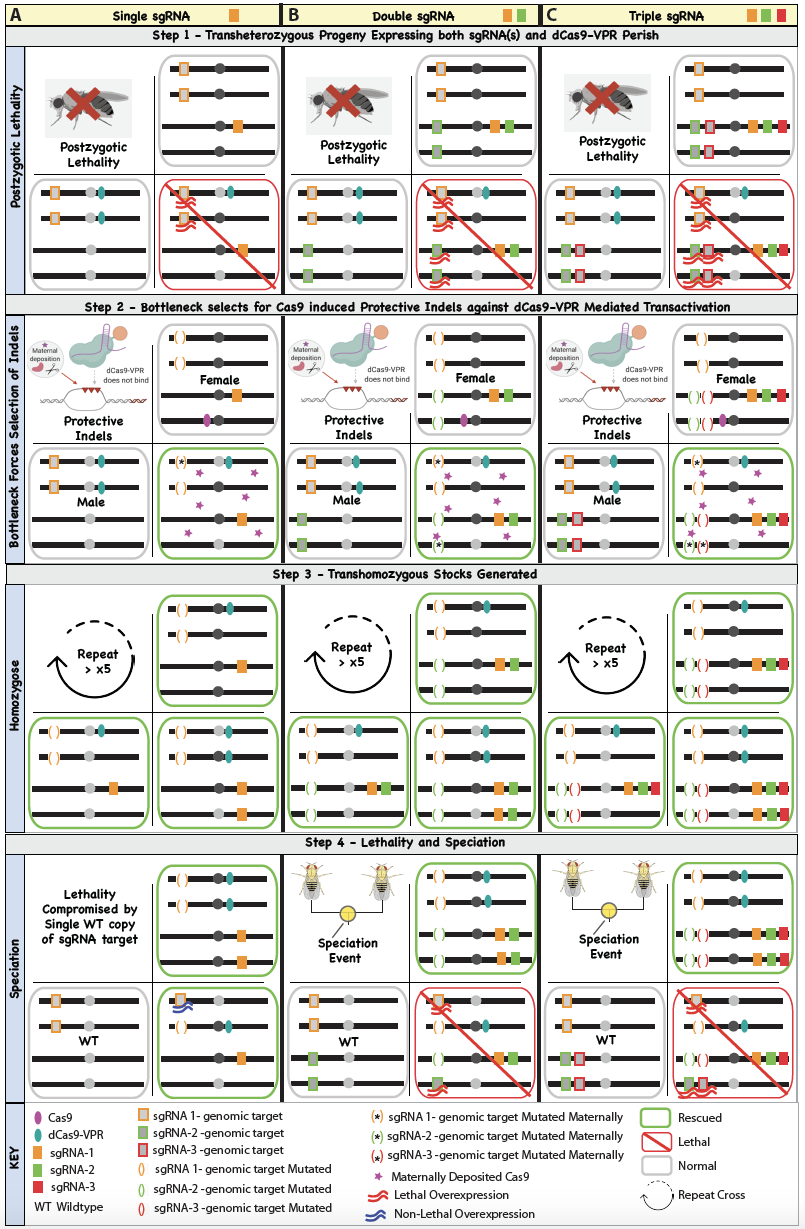
**

**Supplementary Figure 2. Schematic of the genetic crossing scheme used to engineer SPECIES. (A)** Complete lethality (100%) was observed in transheterozygotes (dCas9/+; sgRNA/+) when an sgRNA was crossed to dCas9-VPR due to lethal overexpression (Step 1). To generate protective indels, the sgRNA was first crossed to Cas9, then transheterozygous (Cas9/+; sgRNA/+) females were crossed to dCas9-VPR males generating a bottleneck by which a small proportion of transheterozygotes (dCas9/+; sgRNA/+) survived due to protective indels generated by Cas9/sgRNA (Step 2). Surviving individuals (inheriting Cas9 protein maternally but lacking Cas9 as a gene) were inbred for many generations (>5) to generate homozygous stocks (Step 3). To assess lethality and speciation, homozygous stocks were bidirectionally outcrossed to WT. For a single sgRNA system, complete synthetic lethality and speciation was not observed due to the fact that one WT copy of the target promoter was not sufficient to induce lethal overexpression (A, Step 4). To overcome this issue, we multiplexed using either two sgRNAs **(B),** or three sgRNAs **(C),** and repeated steps 1-4 to engineer reproductively isolated synthetic species.


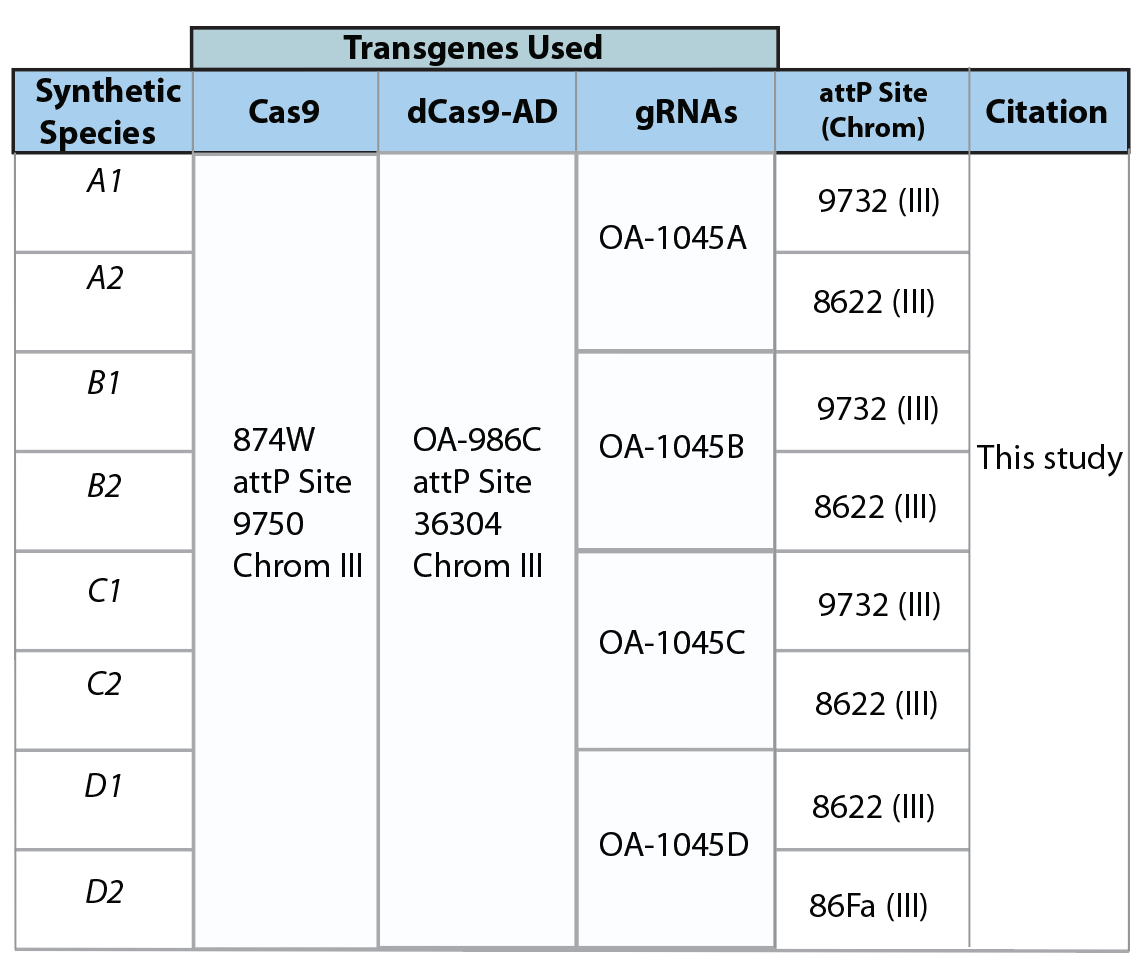


**Supplementary Figure 3. Generation of eight SPECIES.** For each synthetic species (*A1,A2,B1,B2,C1,C2,D1,D2*) the transgene ID, and chromosomal insertion site are listed.

**
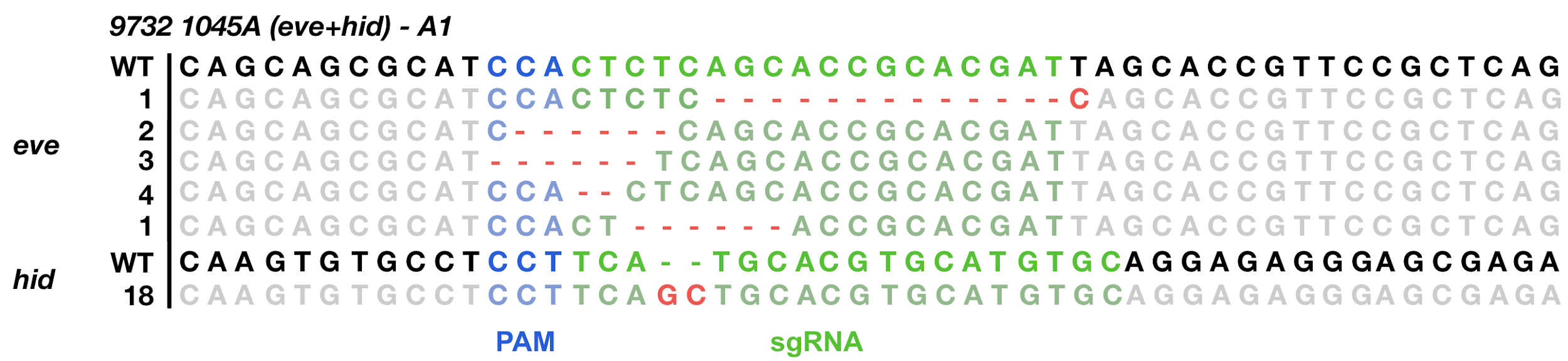
**

**
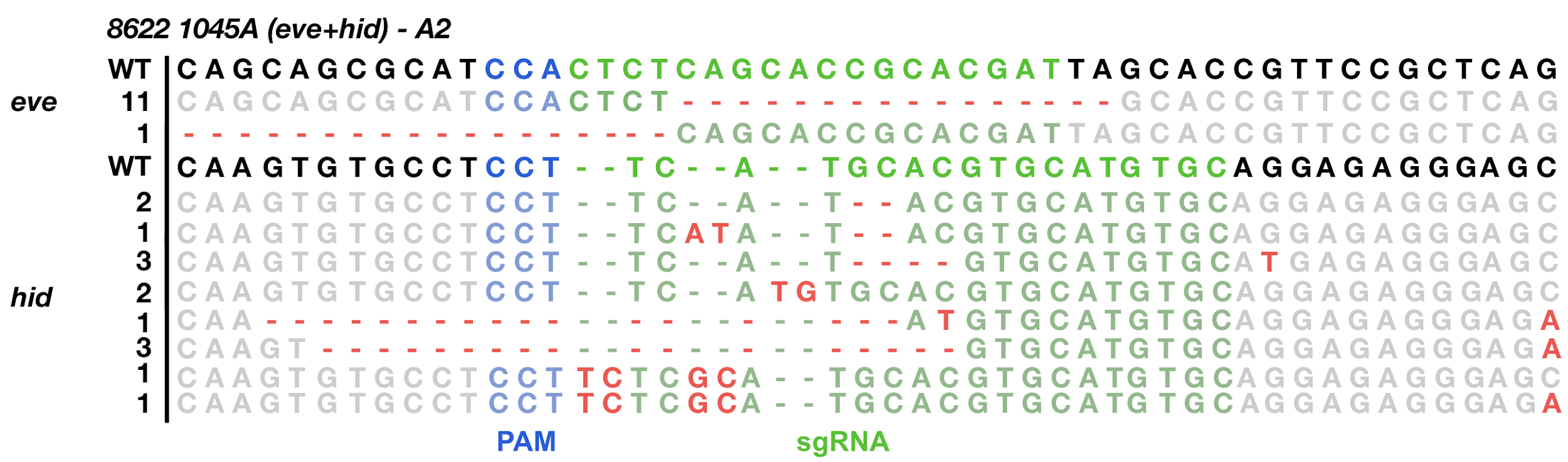
**

**
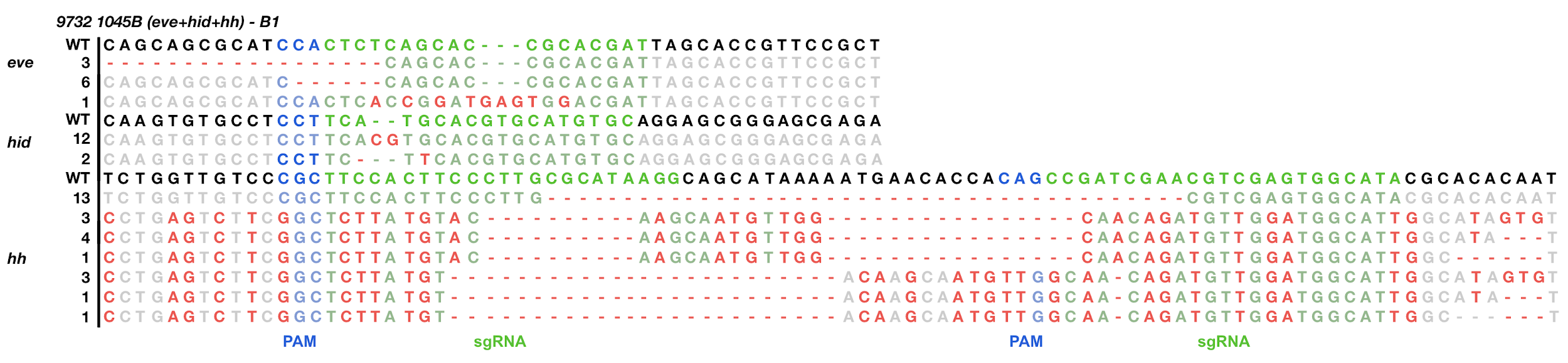
**

**
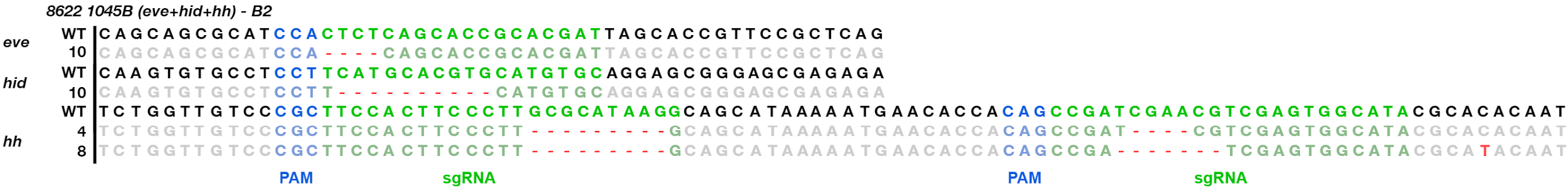
**

**
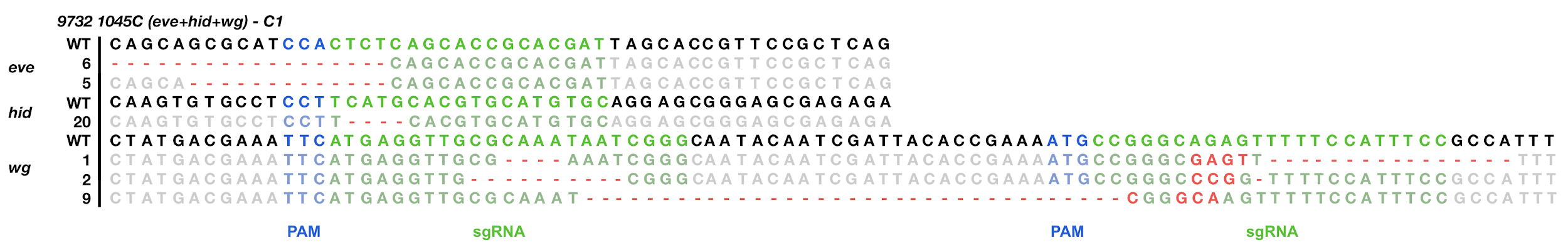
**

**
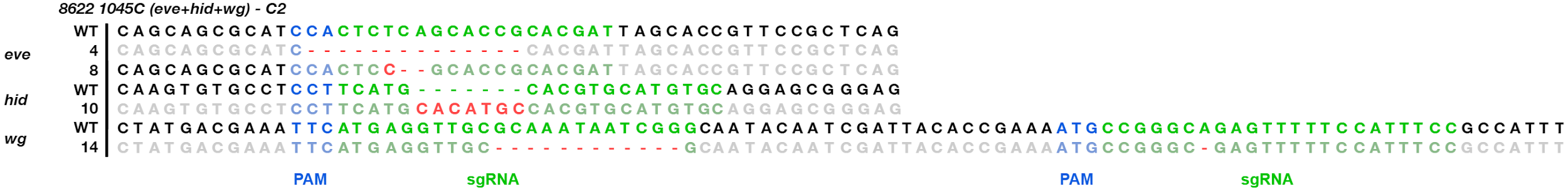
**

**
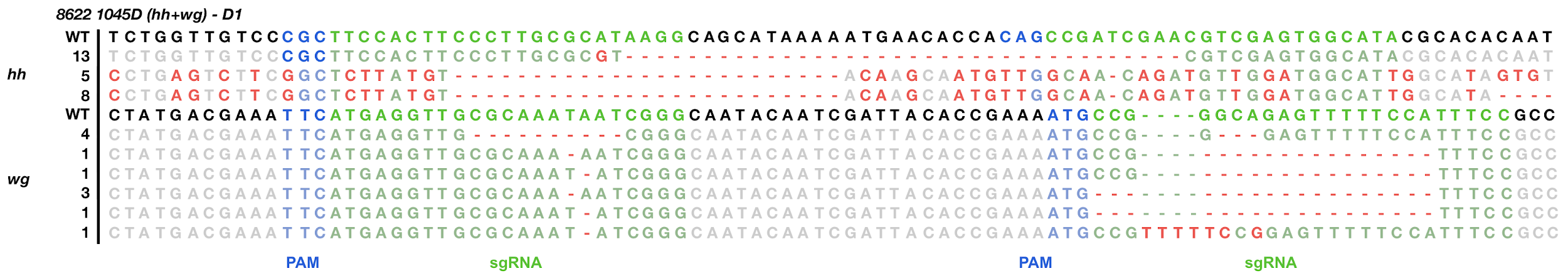
**

**
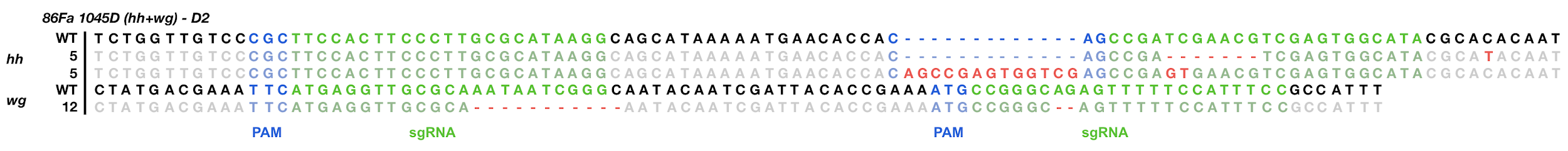
**

**Supplementary Figure 4. Molecular characterization of protective indel mutations.** For the generation of each independent synthetic species (*A1,A2,B1,B2,C1,C2,D1,D2*) the gRNA target site was sanger sequenced and the indels were confirmed. Number to the left of each sequence indicates the number of individuals sequenced with this mutation.

**
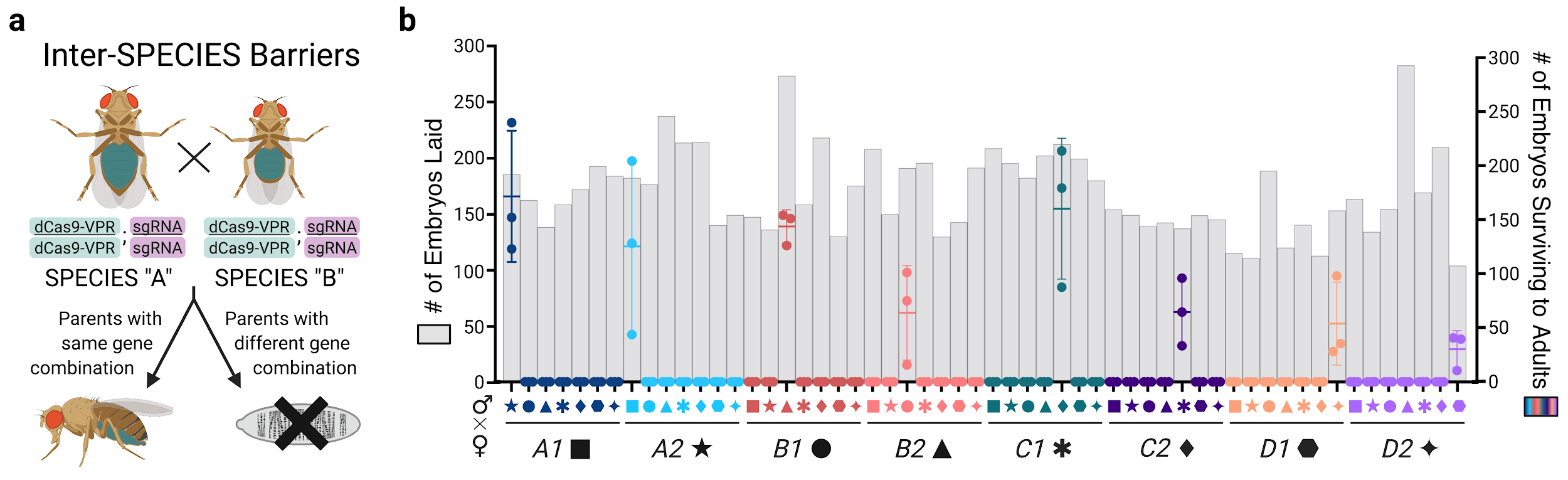
**

**Supplementary Figure 5. Reproductive isolation between double-homozygous speciated lines.** (**a**) Individuals from each SPECIES were crossed to one another to determine the extent of reproductive isolation between SPECIES. (**b**) The total # of embryos laid is plotted in grey as bars on the left y-axis, while the total # of embryos surviving to adults is plotted on the right y-axis as points. N = 3 biologically independent replicates of all eight SPECIES bidirectionally crossed to the remaining seven species. For embryos laid, each bar represents the mean. For embryos surviving to adults, middle lines indicate mean, while error bars represent standard deviation. In the graph, SPECIES “A” is listed by color and there is a symbol indicating SPECIES “B” in the cross (i.e., the star above the *A1* group indicates the cross between an *A1* female and an *A2* male). Source data are provided as a Source Data file.


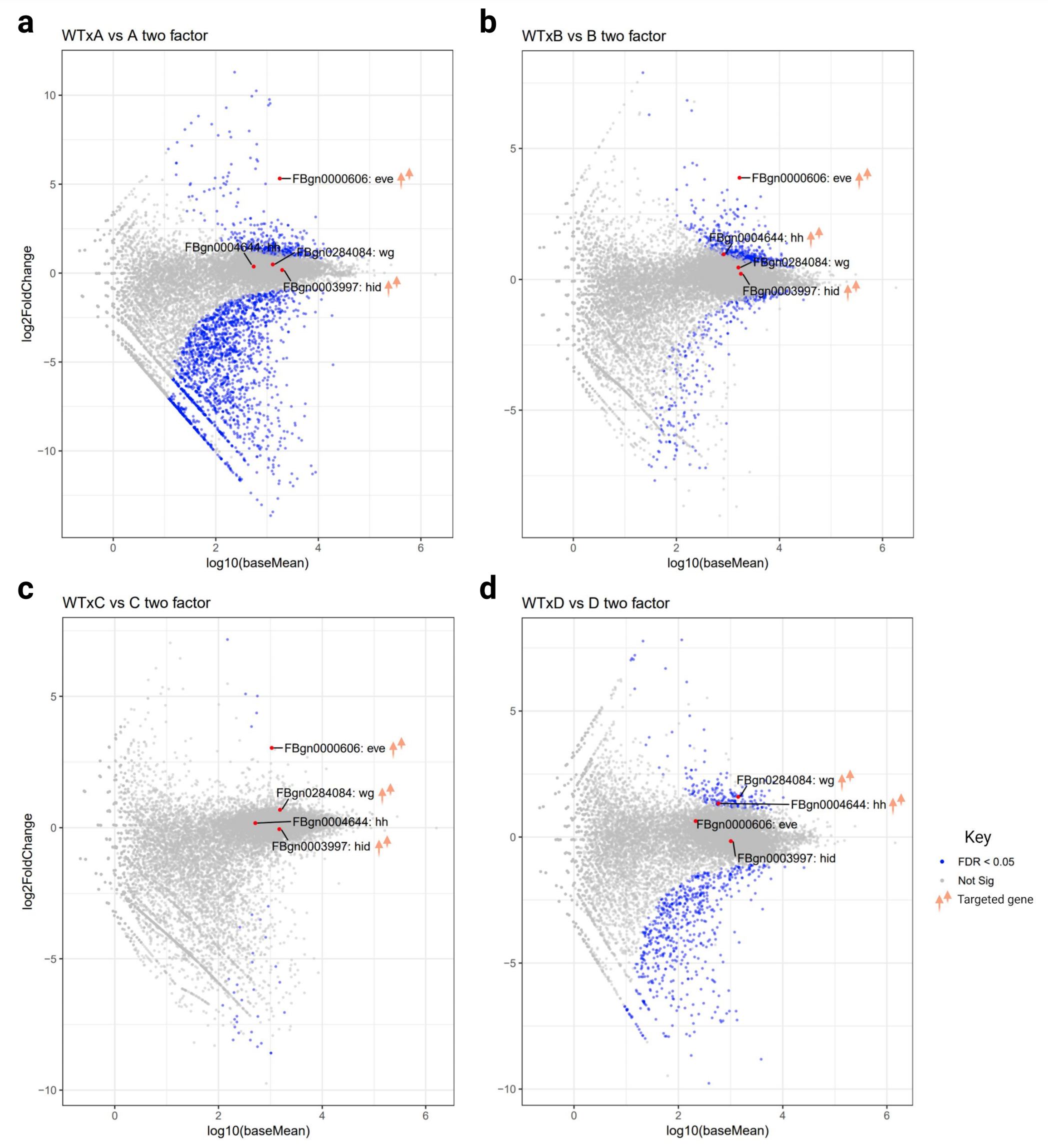


**Supplementary Figure 6. Two-Factor RNAseq comparisons. (a)** Deseq comparisons between RNAseq samples WTx*A1* (sample 8), WTx*A2* (sample 9), *A1*x*A1* (sample 10), *A2*x*A2* (sample 11) observing target/non-target gene misexpression. **(b)** Deseq comparisons between RNAseq samples WTx*B1* (sample 10), WTx*B2* (sample 20), *B1*x*B1* (sample 16) and *B2*x*B2* (sample 22). **(c)** Deseq comparisons between RNAseq samples WTx*C1* (sample 11), WTx*C2* (sample 21), *C1*x*C1* (sample 17), and *C2*x*C2* (sample 23). (**d**) Deseq comparisons between RNAseq samples WTx*D1* (sample 12), WTx*D2* (sample 13), *D1*x*D1* (sample 18), and *D2*x*D2* (sample 19). A full list of RNAseq sample IDs are listed in Supplementary Table 1 and RNAseq data can be found in Supplementary Tables 1-3, 7. Genes that are significantly misexpressed (FDR < 0.05) are colored blue. Source data are provided as a Source Data file.

**
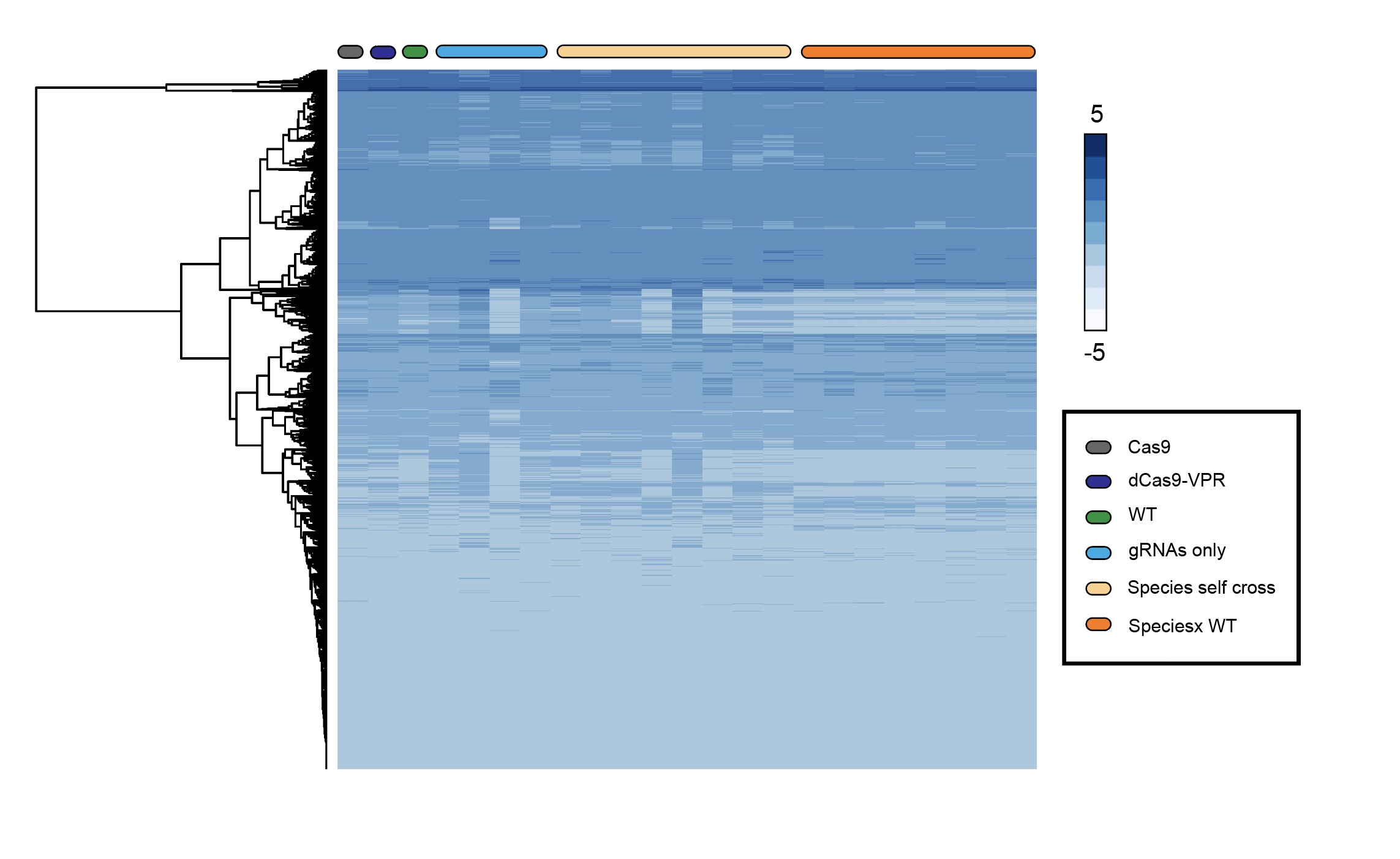
**

**Supplementary Figure 7. Heatmap of the RNAseq Data.** Hierarchical clustering heat map of the RNAseq data (Supplementary Table 2).

**Supplementary Table 5. Primers used in this study.**

| **Primer** | **Primer Sequence, 5’ to 3’** | **Source** |
| --- | --- | --- |
| *attP* sequence  986.C1  986.C2 | CCCACAATGGTTAATTCGAGCTCGCCCGGGTCCTAGGTCGACGATGTAGGTCACGGTCTC  GTTATTTTAAAAACGATTCATTCTAGTTAATTAAGTCGACATGCCCGCCGTGACCGTCGA | plasmid M{3xP3-RFP attP} |
| p10 3’UTR  986.C3  986.C4 | TCGACGGTCACGGCGGGCATGTCGACTTAATTAACTAGAATGAATCGTTTTTAAAATAAC  AAAAGTTGGTGGTGGGGAGGCCACCGAGTATGGGCGCGCCCCGGCCGTTAACTCGAATCG | Addgene plasmid #100580 |
| opie2 promoter fragment  986.C5  986.C6 | GCTGGCTTGGATAGCGATTCGAGTTAACGGCCGGGGCGCGCCCATACTCGGTGGCCTCCC  CCCCGGTGAACAGCTCCTCGCCCTTGCTCACCATCTCGAGCACCAGAGACAGGTTGCGGC | Translocation plasmid B |
| eCFP  986.C7  986.C8 | CGCCATCCAACCGCCGCCGCAACCTGTCTCTGGTGCTCGAGATGGTGAGCAAGGGCGAGG  GTGGTATGGCTGATTATGATCTAGAGTCGCGGCCGCTTACTTGTACAGCTCGTCCATGCC | pJFRC81-10XUAS-IVS-Syn21-GFP-p10 |
| *Ubiquitin-63E* promoter fragment (for 986B)  986.C9  986.C10 | AGCGGGTTCTCGACGGTCACGGCGGGCATGTCGACGCGGCCGCCGCGCAGATCGCCGATG  CAATGGAGTACTTCTTGTCCATGGTGGCAGTTTAAACTCTGCGGGTCAAAATAGAGATGT | *D. melanogaster* genomic DNA |
| dCas9-VPR (for 986B)  986.C11  986.C12 | ATTTTCCACATCTCTATTTTGACCCGCAGAGTTTAAACTGCCACCATGGACAAGAAGTAC  ATTGATTTGTTATTTTAAAAACGATTCATTCTAGTTAATTAATCAAAACAGAGATGTGTC | Addgene plasmid #78898 |
| *bottleneck* promoter fragment (for 986C)  986.C13  986.C14 | GCGGGTTCTCGACGGTCACGGCGGGCATGTCGACGCGGCCGCATTAGATGAACCCCATGG  CCCAATGGAGTACTTCTTGTCCATGGTGGCAGTTTAAACAGCCGAATTCGTTGACGGTTG | *D. melanogaster* genomic DNA |
| dCas9-VPR (for 986C)  986.C15  986.C12 | TTCGTACTTCAACCGTCAACGAATTCGGCTGTTTAAACTGCCACCATGGACAAGAAGTAC  ATTGATTTGTTATTTTAAAAACGATTCATTCTAGTTAATTAATCAAAACAGAGATGTGTC | Addgene plasmid #78898 |
| *Ubiquitin-63E* promoter fragment (for 986D)  986.C9  986.C16 | AGCGGGTTCTCGACGGTCACGGCGGGCATGTCGACGCGGCCGCCGCGCAGATCGCCGATG  TCGTGGCCGCCGGCCTTTTCATGGTGGCAGTTTAAACTCTGCGGGTCAAAATAGAGATGT | *D. melanogaster* genomic DNA |
| dCas9-VP64 (for 986D)  986.C17  986.C18 | TTCCACATCTCTATTTTGACCCGCAGAGTTTAAACTGCCACCATGAAAAGGCCGGCGGCC  ATTGATTTGTTATTTTAAAAACGATTCATTCTAGTTAATTAATTAGCCCTCCCACACATA | Addgene plasmid #78897 |
| *bottleneck* promoter fragment (for 986E)  986.C13  986.C19 | GCGGGTTCTCGACGGTCACGGCGGGCATGTCGACGCGGCCGCATTAGATGAACCCCATGG  TTTCGTGGCCGCCGGCCTTTTCATGGTGGCAGTTTAAACAGCCGAATTCGTTGACGGTTG | *D. melanogaster* genomic DNA |
| dCas9-VP64 (for 986E)  986.C20  986.C18 | GTACTTCAACCGTCAACGAATTCGGCTGTTTAAACTGCCACCATGAAAAGGCCGGCGGCC  ATTGATTTGTTATTTTAAAAACGATTCATTCTAGTTAATTAATCAAAACAGAGATGTGTC | Addgene plasmid #78897 |
| U6:3 promoter fragment  1045.C1  1045.C2 | TTGGGAATTGGGCAATATTTAAATGGCGGCGCGCCGAATTCTTTTTTGCTCACCTGTGAT  CTTATTTTAACTTGCTATTTCTAGCTCTAAAACCCTAGGCCGACGTTAAATTGAAAATAG | Addgene plasmid #49411 |
| sgRNA scaffold  1045.C3  1045.C4 | ATATATAGACCTATTTTCAATTTAACGTCGGCCTAGGGTTTTAGAGCTAGAAATAGCAAG  AGTGGATCTCTAGAGGTACCGTTGCGGCCGCGTTTTAATTAAAAAAGCACCGACTCGGTG | Addgene plasmid #49411 |
| *eve*-sgRNA-U6:1-promoter-*hid*-sgRNA fragment  1045.C5  1045.C6 | GTTCGTATATATAGACCTATTTTCAATTTAACGTCGGATCGTGCGGTGCTGAGAG  CGGACTAGCCTTATTTTAACTTGCTATTTCTAGCTCTAAAACTCATGCACGTGCATGTGC | Custom gBlocks® Gene Fragment |
| *Gypsy*-U6:1-promoter-*hh1*-sgRNA-U6:3-promoter-*hh2*-sgRNA fragment (for OA-1045B)  1045.C7  1045.C8 | GGTGCTTTTTTAATTAAAACGCGGCCGCAACGGTACCTGCAGCCACGTAATAAGTGTGCG  ACACTAGTGGATCTCTAGAACAACTCTCAGGCTCCAGGTAGGCAAAAAAGCACCGACTCG | pCFD-*hh* |
| *Gypsy*-U6:1-promoter-*wg1*-sgRNA-U6:3-promoter-*wg2*-sgRNA fragment (for OA-1045C)  1045.C7  1045.C8 | GGTGCTTTTTTAATTAAAACGCGGCCGCAACGGTACCTGCAGCCACGTAATAAGTGTGCG  ACACTAGTGGATCTCTAGAACAACTCTCAGGCTCCAGGTAGGCAAAAAAGCACCGACTCG | pCFD-*wg* |
| U6:1-promoter-*wg1*-sgRNA-U6:3-promoter-*wg2*-sgRNA fragment (for OA-1045D)  1045.C9  1045.C10 | CCGGGAATTGGGAATTGGGCAATATTTAAATGGCGGCGCGCCAGCCGATCAATTGAGATC  TGTTTTTGCGAATAAATTCAACGCACACTTATTACGTGCATATGAACAACTCTCAGGCTC | pCFD-*wg* |
| *Gypsy*-U6:1-promoter-*hh1*-sgRNA-U6:3-promoter-*hh2*-sgRNA fragment (for OA-1045D)  1045.C11  1045.C12 | CTGGAGCCTGAGAGTTGTTCATATGCACGTAATAAGTGTGCGTTG  TTTATTGAACAACTCTCAGGCTCCAGGTAGTCTAGAGCAAAAAAGCACCGACTCGGTGCC | pCFD-*hh* |
| tRNA-*eve*-sgRNA-tRNA-*hid*-sgRNA-tRNA-*hh1*-sgRNA-tRNA-*hh2*-sgRNA-U6:3 UTR fragment (for OA-1045E)  1045.C13  1045.C14 | TCGTATATATAGACCTATTTTCAATTTAACGTCGGTTAATTAAGGGCTTTGAGTGTGTGT  TCGTCGACACTAGTGGATCTCTAGAGGTACCGTTGCGGCCGCATGCATACGCATTAAGCG | Gene synthesized vector |
