# Supplementary material for: Engineered reproductively isolated species drive reversible population replacement
Source: Nat Commun. 2021 Jun 2;12:3281. doi: 10.1038/s41467-021-23531-z (PMC8173020; doi:10.1038/s41467-021-23531-z)
Supplement: Supplementary file 3 — Reporting Summary [file 41467_2021_23531_MOESM3_ESM.pdf]

nature research

Corresponding author(s): Omar Akbari

Last updated by author(s): 2021 0422

Reporting Summary

Nature Research wishes to improve the reproducibility of the work that we publish. This form provides structure for consistency and transparency in reporting. For further information on Nature Research policies, see our Editorial Policies and the Editorial Policy Checklist.

Statistics

For all statistical analyses, confirm that the following items are present in the figure legend, table legend, main text, or Methods section.

n/a	Confirmed	
☐	☒	The exact sample size (*n*) for each experimental group/condition, given as a discrete number and unit of measurement
☐	☒	A statement on whether measurements were taken from distinct samples or whether the same sample was measured repeatedly
☐	☒	The statistical test(s) used AND whether they are one- or two-sided<br>*Only common tests should be described solely by name; describe more complex techniques in the Methods section.*
☒	☐	A description of all covariates tested
☒	☐	A description of any assumptions or corrections, such as tests of normality and adjustment for multiple comparisons
☐	☒	A full description of the statistical parameters including central tendency (e.g. means) or other basic estimates (e.g. regression coefficient) AND variation (e.g. standard deviation) or associated estimates of uncertainty (e.g. confidence intervals)
☐	☒	For null hypothesis testing, the test statistic (e.g. *F*, *t*, *r*) with confidence intervals, effect sizes, degrees of freedom and *P* value noted<br>*Give P values as exact values whenever suitable.*
☐	☒	For Bayesian analysis, information on the choice of priors and Markov chain Monte Carlo settings
☐	☒	For hierarchical and complex designs, identification of the appropriate level for tests and full reporting of outcomes
☐	☒	Estimates of effect sizes (e.g. Cohen's *d*, Pearson's *r*), indicating how they were calculated

Our web collection on statistics for biologists contains articles on many of the points above.

Software and code

Policy information about availability of computer code

Data collection	N/A
Data analysis	Code used to analyze drive experiment data and generate Figures 4-6 is available on GitHub at https://github.com/Marshalllab/SPECIES. Illumina Reads were mapped to the Drosophila melanogaster genome (BDGP release 6, Gen Bank accession GCA_000001 215.4) using STAR aligner 2.7.6a. Base calls were performed with RTA 1.18.64 followed by conversion to FASTQ with bcl2fastq 1.8.4. Data was analyzed with GraphPad Prism version 8.2.1 for Windows (GraphPad Software, San Diego CA, www.graphpad.com).

For manuscripts utilizing custom algorithms or software that are central to the research but not yet described in published literature, software must be made available to editors and reviewers. We strongly encourage code deposition in a community repository (e.g. GitHub). See the Nature Research guidelines for submitting code & software for further information.

Data

Policy information about availability of data

All manuscripts must include a data availability statement. This statement should provide the following information, where applicable:

- Accession codes, unique identifiers, or web links for publicly available datasets
- A list of figures that have associated raw data
- A description of any restrictions on data availability

The RNA sequencing data generated in this study is available at NCBI SRA under accession number PRJNA578541 (https://www.ncbi.nlm.nih.gov/bioproject/PRJNA578541). All plasmids and annotated DNA sequence maps are available at www.addgene.com under accession numbers; 112686 (https://www.addgene.org/112686/); 124999 (https://www.addgene.org/124999/); 125000 (https://www.addgene.org/125000/); 125001 (https://www.addgene.org/125001/); 125002 (https://www.addgene.org/125002/); 125003 (https://www.addgene.org/125003/); 125004 (https://www.addgene.org/125004/); 125005 (https://www.addgene.org/125005/); 125006 (https://www.addgene.org/125006/); 125007 (https://

www.addgene.org/125007/). The fly strains engineered in this study and used to generate SPECIES A1 will be available at the Bloomington fly stock center with the stock numbers 79005 (https://bdsc.indiana.edu/Home/Search?presearch=79005) , 91792 (https://bdsc.indiana.edu/Home/Search?presearch=91792) , 91791 (https://bdsc.indiana.edu/Home/Search?presearch=91791).SPECIES fly lines will be made available from the corresponding author upon reasonable request upon agreement to suggested guidelines for the laboratory confinement of gene-drive systems 13,44.

Field-specific reporting

Please select the one below that is the best fit for your research. If you are not sure, read the appropriate sections before making your selection.

- [x] Life sciences
- [ ] Behavioural & social sciences
- [ ] Ecological, evolutionary & environmental sciences

For a reference copy of the document with all sections, see nature.com/documents/nr-reporting-summary-flat.pdf

Life sciences study design

All studies must disclose on these points even when the disclosure is negative.

Sample size	At least three biological replicates were performed per experiment. These sample sizes are sufficient as we performing genetic experiments
Data exclusions	No data was excluded
Replication	At least three biological replicates were performed per experiment, and each experiment was successful.
Randomization	Flies were blindly chosen at random
Blinding	Flies were blindly chosen at random

Reporting for specific materials, systems and methods

We require information from authors about some types of materials, experimental systems and methods used in many studies. Here, indicate whether each material, system or method listed is relevant to your study. If you are not sure if a list item applies to your research, read the appropriate section before selecting a response.

Materials & experimental systems

n/a	Involved in the study	
[ ]	[x]	Antibodies
[x]	[ ]	Eukaryotic cell lines
[x]	[ ]	Palaeontology and archaeology
[ ]	[x]	Animals and other organisms
[x]	[ ]	Human research participants
[x]	[ ]	Clinical data
[x]	[ ]	Dual use research of concern

Methods

n/a	Involved in the study	
[x]	[ ]	ChIP-seq
[x]	[ ]	Flow cytometry
[x]	[ ]	MRI-based neuroimaging

Antibodies

Antibodies used	For antibody staining, embryos were collected overnight and then fixed and dechorionated using standard protocols 38. We used guinea pig anti-Runt polyclonal antibody (kindly provided by David Kosman and John Reinitz) at a concentration of 1:200 and mouse anti-Eve monoclonal 3C10 (developed by C. Goodman and available from the Developmental Studies Hybridoma Bank) at 1:20. Nuclei were counterstained with DAPI. Embryos were stained using standard protocols 39.
Validation	used to stain control embryos.

Animals and other organisms

Policy information about studies involving animals; ARRIVE guidelines recommended for reporting animal research

Laboratory animals	Drosophila melanogaster, both males and females, age included all stages of development. We also used five Global Diversity Lines (from Beijing, China; Ithaca, NY; the Netherlands; Tasmania, Australia; and Zimbabwe, Africa).
Wild animals	No wild animals were used in this study
Field-collected samples	No Field-collected samples were used in this study

Ethics oversight	We have complied with all relevant ethical regulations for animal testing and research and conformed to the UCSD institutionally approved biological use authorization protocol (BUA #R2401).

Note that full information on the approval of the study protocol must also be provided in the manuscript.
