# Supplementary material for: Engineered reproductively isolated species drive reversible population replacement
Source: Nat Commun. 2021 Jun 2;12:3281. doi: 10.1038/s41467-021-23531-z (PMC8173020; doi:10.1038/s41467-021-23531-z)
Supplement: Supplementary file 4 — Description of Additional Supplementary Files [file 41467_2021_23531_MOESM4_ESM.pdf]

Title: Supplementary Video 1.

Description: Animation on engineering SPECIES. A video explaining the process of how to generate SPECIES, covering CRISPR gene editing, the CRISPRbased transactivation, the self-cross and outcross experiment, potential applications, and significant advance. Embryos from twenty four independent genetic crosses consisting of each SPECIES (A1, A2, B1, B2, C1, C2, D1, D2) either self-crossed (SPECIES ♀ X SPECIES ♂), or bidirectionally out-crossed to WT (SPECIES♀ X WT♂ or SPECIES ♂ X WT♀) were laid on grape plates for 12 hours (# of embryos ranging from 27-135) . Zoomed in images covering ~20% of the grape plate were taken once per day for 5 days. Embryo survival can be seen for each SPECIES when selfcrossed but not when out-crossed to WT due to reproductive isolation.
